# Quantum Estimates for Different Type Intequalities through Generalized Convexity

**DOI:** 10.3390/e24050728

**Published:** 2022-05-20

**Authors:** Ohud Bulayhan Almutairi

**Affiliations:** Department of Mathematics, University of Hafr Al-Batin, Hafr Al-Batin 31991, Saudi Arabia; dr.ohudalmutairi@uhb.edu.sa

**Keywords:** *q*-integral inequalities, (*h* − *m*)-convex function, Hermite-Hadamard inequality, Simpson’s inequality, quantum calculus

## Abstract

This article estimates several integral inequalities involving (h−m)-convexity via the quantum calculus, through which Important integral inequalities including Simpson-like, midpoint-like, averaged midpoint-trapezoid-like and trapezoid-like are extended. We generalized some quantum integral inequalities for q-differentiable (h−m)-convexity. Our results could serve as the refinement and the unification of some classical results existing in the literature by taking the limit $q\to 1^{-}$.

## 1. Introduction

Quantum Calculus, simply represented by *q*-calculus, deals with the study of calculus without limits. Thus, the *q*-analogues of mathematical objects can be recaptured through $q\to 1^{-}$. This area of study was apparently first discovered by Euler in the eighteenth century where the parameter *q* was introduced in the finite series studied by Newton.

Quantum Calculus has attracted the attention of many researchers. This is due to the numerous applications of the field in different areas of sciences such as combinatorics, mechanics, orthogonal polynomials. Other applications include the quantum information theory, an interesting area with many branches like computer science, cryptography and philosophy [1,2,3]. For example, both *q*-derivative and *q*-integral of a function were defined on a finite interval, and the results obtained were applied to determine the existence and uniqueness solution of *q*-impulsive equations diffrence equtions [4]. Others include the construction of a q-deformed variant for the conformal quantum mechanics models—with a complex deformation parameter, and the differential calculus associated with such quantity was equally studied [5].

Considering these vast applications, many researchers extend the concept of *q*-calculus to incorporate different integral inequalities including Newton’s, Simpson’s, Ostrowski, Hölder, Grüss Simpson’s, Grüss-Čebyšev, Hermite-Hadamard, Cauchy-Bunyakovsky-Schwarz inequalities [6,7,8,9]. These can be studied through classical convexity as well as their generalizations, such as *m*-convexity, *s*-convexity and preinvex functions [10,11,12].

In the literature, many studies focused on *q*-analogues of midpoint type, trapezoidal type and Ostrowski’s type inequalities for *q*-differentiable variant type of convex functions [13,14,15,16]. For example, Sudsutad et al. [17] established new *q*-analogues of trapezoid-like for *q* differentiable convex functions. A variant type of midpoint-like and trapezoid-like of quantum inequalities for (α, m)-convexity have been studied [18]. Others include the work of Dafang Zhao et al. [15] who established some quantum inequalities of midpoint and trapezoidal type via quasi-convexity.

Motivated by the aforementioned literature, this study is concerned with establishing variant types of q-integral inequalities for (h,m)-convexity, associated with Simpson-like, midpoint-like, trapezoid-like and averaged midpoint-trapezoid-like types inequalities. We equally obtained some results—by choosing a special parameter value—that are relevant with those reported in previous studies.

The rest of this paper is organized as follows: Section 2 presents the preliminary studies. While Section 3 generalizes several *q*-integral inequalities via (h−m)-convexity, Section 4 discusses different special cases of the main results. The study is concluded in Section 5.

## 2. Preliminaries

This section presents some important definitions in the *q*-calculus along with inequalities related to them. These Preliminary results would later in this study help us prove some of our results. We first begin with the following notation [2]:

$${\left[s\right]}_{q}=\left(1-q^{s}\right).\frac{1}{1-q}=1+q+q^{2}+\cdots +q^{s-1},\;0<q<1.$$

The *q*-Jackson integral of a mapping A from 0 to *c* is defined follows [19]:

$$\int_{r}^{c}A\left(\theta \right)d_{q}\theta =\int_{0}^{c}A\left(\theta \right)d_{q}\theta -\int_{0}^{r}A\left(\theta \right)d_{q}\theta$$

**Definition** **1** ([20])**.** *Assume that A:J→R is a continuous mapping and θ∈J. Then the q-derivative on J of A at θ is given as*

$${}_{r}D_{q}A\left(\theta \right)=\frac{A(\theta )-A(q\theta +(1-q)r)}{\left(1-q)(\theta -r\right)},\;\theta \neq r,\;{}_{r}D_{q}A\left(r\right)=\lim_{\theta \to r}{}_{r}D_{q}A\left(\theta \right)$$

**Definition** **2** ([20])**.** *Assume that A:J→R is a continuous mapping. Then the q-integral on J is given as*

$$\int_{r}^{m}A{\left(\theta \right)}_{r}\;d_{q}\theta =\left(1-q\right)\left(z-r\right)\sum_{s=0}^{\infty }q^{s}A\left(q^{s}z+\left(1-q^{s}\right)r\right)$$

*for z∈I. Moreover, if c∈(r,z), then the q-integral on J is given as*

$$\int_{c}^{m}A{\left(\theta \right)}_{r}d_{q}\theta =\int_{r}^{z}A{\left(\theta \right)}_{r}\;d_{q}\theta -\int_{r}^{c}A{\left(\theta \right)}_{r}\;d_{q}\theta$$

**Definition** **3** ([20])**.** *Assume that A:[r,c]→R is a mapping. The $q_{r}$-definite integral on [r,c] is defined as follows*

$$\begin{array}{cc} \int_{r}^{c}A{\left(\theta \right)}_{r}d_{q}\theta & =\left(1-q\right)\left(c-r\right)\sum_{s=0}^{\infty }q^{s}A\left(q^{s}c+\left(1-q^{s}\right)r\right)\\ & =\left(c-r\right)\int_{0}^{1}A\left(\left(1-\theta \right)r+\theta c\right)d_{q}\theta \end{array}$$

Following the above definitions, the Hölder inequality was studied in the setup of *q*-calculus

**Theorem** **1** ([20])**.** *Suppose that A,W:I→R are two continuous mappings. The following*

$$\int_{r}^{x}{\left|A\left(\theta \right)||W\left(\theta \right)\right|}_{r}\;d_{q}\theta \leq {\left(\int_{r}^{x}{\left|A\left(\theta \right)\right|}^{v_{1}}{}_{r}\;d_{q}\theta \right)}^{\frac{1}{v_{1}}}{\left(\int_{r}^{x}{\left|W\left(\theta \right)\right|}^{v_{2}}{}_{r}\;d_{q}\theta \right)}^{\frac{1}{v_{2}}}$$

*holds for all x∈I and $v_{1},v_{2}>1$ with $v_{1}^{-1}+v_{2}^{-1}=1$.*

Using the q-calculus, Alp et al. [21] improved the Hermite-Hadamard inequality as follows.

**Theorem** **2.** *Suppose that A:J→R is convex and differentiable on J with q∈(0,1). Then the following*

$$A\left(\frac{qr+c}{1+q}\right)\leq \frac{1}{c-r}\int_{r}^{c}A{\left(\theta \right)}_{r}\;d_{q}\theta \leq \frac{qA(r)+A(c)}{1+q}$$

*holds.*

The following theorem present the *q*-midpoint type inequality via convexity.

**Theorem** **3** ([21])**.** *Suppose that A:[r,c]→R is a q-differentiable function on (r,c), ${}_{r}D_{q}A$ be continuous and integrable on [r,c] and 0<q<1. If $\left|{}_{r}D_{q}A\right|$ is convex on [r,c], then the following q-midpoint type inequality*

$$\begin{array}{c} \left|A\left(\frac{qr+c}{1+q}\right)-\frac{1}{\left(c-r\right)}\int_{r}^{c}A{\left(\theta \right)}_{r}d_{q}\theta \right|\\ ⩽q\left(c-r\right)\left[\left|{}_{r}D_{q}A\left(c\right)\right|\frac{3}{{\left(1+q\right)}^{3}\left(1+q+q^{2}\right)}+\left|{}_{r}D_{q}A\left(r\right)\right|\frac{-1+2q+2q^{2}}{{\left(1+q\right)}^{3}\left(1+q+q^{2}\right)}\right] \end{array}$$

*holds.*

The new *q*-integral inequalities for (α,m)-convexity werer studied by Zhang et al. [18] through the following lemma

**Lemma** **1.** *Assume that A:J→R is a continuous and q-differentiable mapping on $J^{\circ }$ with q∈(0,1). Then the following*

$$\begin{array}{c} \gamma_{1}\left[\gamma_{2}A\left(c\right)+\left(1-\gamma_{2}\right)A\left(r\right)\right]+\left(1-\gamma_{1}\right)A\left(\gamma_{2}c+\left(1-\gamma_{2}\right)r\right)-\frac{1}{c-r}\int_{r}^{c}A\left(x\right){}_{r}d_{q}x\\ =\left(c-r\right)\left\{\int_{0}^{\gamma_{2}}{\left(q\theta +\gamma_{1}\gamma_{2}-\gamma_{1}\right)}_{r}D_{q}A{\left(\theta c+\left(1-\theta \right)r\right)}_{0}\;d_{q}\theta \right.\\ \left.\;+\int_{\gamma_{2}}^{1}{\left(q\theta +\gamma_{1}\gamma_{2}-1\right)}_{r}D_{q}A{\left(\theta c+\left(1-\theta \right)r\right)}_{0}\;d_{q}\theta \right\} \end{array}$$

*holds for all $\gamma_{1},\gamma_{2}\in \left[0,1\right]$ if ${}_{r}D_{q}A$ is integrable on I*

In [22], the authors introduced the definition of the class (h,m)-convexity as

**Definition** **4.** *Let h:I⊆R→R be a positive function. The function A:[0,b]→R is called (h−m) convex function, if A is positive and*

(1) A(θr+m(1−θ)c)≤h(γ)A(r)+mh(1−θ)A(c)

*for all r,c∈[0,b],m∈[0,1] and θ∈(0,1).*

If the inequality (1) is reversed, then A is said to be (h−m)-concave mapping on [0,b].

## 3. Mian Result

In this section, we give generalization of quantum integral inequalities for (h−m)-convexity of the absolute values of the *q*-derivative.

**Theorem** **4.** *For 0≤r<c and m∈(0,1], assume that $A:\left[r,\frac{c}{m}\right]\to R$ is a continuous and q-differentiable mapping on $\left(r,\frac{c}{m}\right)$. Let ${}_{r}D_{q}A$ be integrable on $\left[r,\frac{c}{m}\right]$ with 0<q<1, then the inequality*

(2) $$\begin{array}{c} \left|\gamma_{1}\left[\gamma_{2}A\left(c\right)+\left(1-\gamma_{2}\right)A\left(r\right)\right]+\left(1-\gamma_{1}\right)A\left(\gamma_{2}c+\left(1-\gamma_{2}\right)r\right)-\frac{1}{c-r}\int_{r}^{c}A\left(x\right){}_{r}d_{q}x\right|\\ \leq \left(c-r\right)\left\{\left[\int_{0}^{\gamma_{2}}h\left(\theta \right)|q\theta -\left(\gamma_{1}-\gamma_{1}\gamma_{2}\right)|{}_{0}d_{q}\theta +\int_{0}^{1}h\left(\theta \right)\left|q\theta -\left(1-\gamma_{1}\gamma_{2}\right)\right|{}_{0}d_{q}\theta \right.\right.\\ \left.-\int_{0}^{\gamma_{2}}h\left(\theta \right){\left|q\theta -\left(1-\gamma_{1}\gamma_{2}\right)\right|}_{0}\;d_{q}\theta \right]\left|{}_{r}D_{q}A\left(c\right)\right|\\ +m\left[\int_{0}^{\gamma_{2}}h\left(1-\theta \right)|q\theta -\left(\gamma_{1}-\gamma_{1}\gamma_{2}\right)|{}_{0}d_{q}\theta +\int_{0}^{1}h\left(1-\theta \right)\left|q\theta -\left(1-\gamma_{1}\mu \right)\right|{}_{0}d_{q}\theta \right.\\ -\int_{0}^{\gamma_{2}}h\left(1-\theta \right)\left|q\theta -\left(1-\gamma_{1}\gamma_{2}\right)\right|{}_{0}d_{q}\theta ]\left|{}_{r}D_{q}A\left(\frac{r}{m}\right)|\right\} \end{array}$$

*holds for all $\gamma_{1},\gamma_{2}\in \left[0,1\right]$ if ${}_{r}D_{q}f$ is (h−m)-convex on $\left[r,\frac{c}{m}\right]$.*

**Proof.**  Applying the (h,m)-convexity of $\left|{}_{r}D_{q}A\right|$, property of the modulus and Lemma 1, we obtain

$$\begin{array}{cc} |\gamma_{1}[ & \gamma_{2}A\left(c\right)+\left(1-\gamma_{2}\right)A\left(r\right)]+\left(1-\gamma_{1}\right)A\left(\gamma_{2}c+\left(1-\gamma_{2}\right)r\right)-\frac{1}{c-r}\int_{r}^{c}A{\left(x\right)}_{r}\;d_{q}x|\\ \leq & \left(c-r\right)\left\{\left.\left.\int_{0}^{\gamma_{2}}\left|q\theta +\gamma_{1}\gamma_{2}-\gamma_{1}\right|\right|{}_{r}D_{q}A\left(\theta c+\left(1-\theta \right)r\right)\right|{}_{0}d_{q}t\right.\\ & \left.+\left.\left.\int_{\gamma_{2}}^{1}\left|q\theta +\gamma_{1}\gamma_{2}-1\right|\right|{}_{r}D_{q}A\left(\theta c+\left(1-\theta \right)r\right)\right|{}_{0}d_{q}t\right\}\\ \leq & \left(c-r\right)\left\{\int_{0}^{\gamma_{2}}\left|q\theta -\left(\gamma_{1}-\gamma_{1}\gamma_{2}\right)\right|\left[h\left(\theta \right)\left|{}_{r}D_{q}A\left(c\right)\right|+mh\left(1-\theta \right)\left|{}_{r}D_{q}A\left(\frac{r}{m}\right)\right|\right]{}_{0}d_{q}\theta \right.\\ & \left.+\int_{\gamma_{2}}^{1}\left|q\theta -\left(1-\gamma_{1}\gamma_{2}\right)\right|\left[h\left(\theta \right)|{}_{r}D_{q}A\left(c\right)\left|+mh(1-\theta )\right|{}_{r}D_{q}A\left(\frac{r}{m}\right)|\right]{}_{0}d_{q}\theta \right\}\\ = & \left(c-r\right)\left\{\int_{0}^{\gamma_{2}}\left|q\theta -\left(\gamma_{1}-\gamma_{1}\gamma_{2}\right)\right|\left[h\left(\theta \right)\left|{}_{r}D_{q}A\left(c\right)\right|+{\left.mh(1-\theta )\right|}_{r}D_{q}A\left(\frac{r}{m}\right)|\right]{}_{0}d_{q}\theta \right.\\ & +\int_{0}^{1}\left|q\theta -\left(1-\gamma_{1}\gamma_{2}\right)\right|\left[{\left.h(\theta )\right|}_{r}D_{q}A\left(c\right)\left|+mh(1-\theta )\right|{}_{r}D_{q}A\left(\frac{r}{m}\right)|\right]{}_{0}d_{q}t\\ & \left.-\int_{0}^{\gamma_{2}}\left|q\theta -\left(1-\gamma_{1}\gamma_{2}\right)\right|\left[h\left(\theta \right)\left|{}_{r}D_{q}A\left(c\right)\right|+mh\left(1-\theta \right)\left|{}_{r}D_{q}A\left(\frac{r}{m}\right)\right|\right]{}_{0}d_{q}\theta \right\}\\ = & \left(c-r\right)\left\{\left[\int_{0}^{\gamma_{2}}h\left(\theta \right)|q\theta -\left(\gamma_{1}-\gamma_{1}\gamma_{2}\right)|{}_{0}d_{q}t+\int_{0}^{1}h\left(\theta \right)\left|q\theta -\left(1-\gamma_{1}\gamma_{2}\right)\right|{}_{0}d_{q}\theta \right.\right.\\ & \left.-\int_{0}^{\gamma_{2}}h\left(\theta \right){\left|q\theta -\left(1-\gamma_{1}\gamma_{2}\right)\right|}_{0}\;d_{q}\theta \right]\left|{}_{r}D_{q}A\left(c\right)\right|\\ & +m\left[\int_{0}^{\gamma_{2}}h\left(1-\theta \right)|q\theta -\left(\gamma_{1}-\gamma_{1}\gamma_{2}\right)|{}_{0}d_{q}\theta +\int_{0}^{1}h\left(1-\theta \right)\left|q\theta -\left(1-\gamma_{1}\gamma_{2}\right)\right|{}_{0}d_{q}\theta \right.\\ & -\int_{0}^{\gamma_{2}}h\left(1-\theta \right)\left|q\theta -\left(1-\gamma_{1}\gamma_{2}\right)\right|{}_{0}d_{q}\theta ]\left|{}_{r}D_{q}A\left(\frac{r}{m}\right)|\right\}. \end{array}$$

□

If $|{}_{r}D_{q}A{|}^{\mu }$ for μ≥1 is (h,m)-convex, then we can obtain the following result.

**Theorem** **5.** *Assume that $A:\left[r,\frac{c}{m}\right]\to R$ is a continuous and q-differentiable function on $\left(r,\frac{c}{m}\right)$, such that 0≤r<c and m∈(0,1]. Assume that ${}_{r}D_{q}A$ is integrable on $\left[r,\frac{c}{m}\right]$, with 0<q<1, the inequality*

$$\begin{array}{cc} |\gamma_{1}[ & \gamma_{2}A\left(c\right)+\left(1-\gamma_{2}\right)A\left(r\right)]+\left(1-\gamma_{2}\right)A\left(\gamma_{2}c+\left(1-\gamma_{2}\right)r\right)-\frac{1}{c-r}\int_{r}^{c}A{\left(x\right)}_{r}\;d_{q}x|\\ \leq & \left(c-r\right)\left\{{\left[\int_{0}^{1}{\left|q\theta -\left(1-\gamma_{1}\gamma_{2}\right)\right|}_{0}\;d_{q}\theta \right]}^{1-\frac{1}{\mu }}\right.\\ & \times \left[\left(\int_{0}^{1}h\left(\theta \right)\left|q\theta -\left(1-\gamma_{1}\gamma_{2}\right)\right|{}_{0}d_{q}\theta \right){\left|rD_{q}A\left(c\right)\right|}^{\mu }\right.\\ & {\left.+m\left(\int_{0}^{1}h\left(1-\theta \right)\left|q\theta -\left(1-\gamma_{1}\gamma_{2}\right)\right|{}_{0}d_{q}\theta \right){\left|rD_{q}A\left(\frac{r}{m}\right)\right|}^{\mu }\right]}^{\frac{1}{\mu }}\\ & +\left(1-\gamma_{1}\right)\gamma_{2}^{1-\frac{1}{\mu }}\left[\left(\int_{0}^{\gamma_{2}}h\left(\theta \right){}_{0}d_{q}\theta \right){\left|{}_{r}D_{q}A\left(c\right)\right|}^{\mu }\right.\\ & \left.{\left.+{\left.\left.m\left(\int_{0}^{\gamma_{2}}h\left(1-\theta \right){}_{0}d_{q}\theta \right)\right|{}_{r}D_{q}A\left(\frac{r}{m}\right)\right|}^{\mu }\right]}^{\frac{1}{\mu }}\right\} \end{array}$$

*holds for all $\gamma_{1},\gamma_{2}\in \left[0,1\right]$ if ${\left|{}_{r}D_{q}A\right|}^{\mu }$ for μ≥1 is (h,m)-convex on $\left[r,\frac{c}{m}\right]$.*

**Proof.**  Since ${|}_{\mu }D_{q}A{|}^{\mu }$ is (h,m)-convexity, we get

(3) $$\begin{array}{cc} \int_{0}^{1}| & q\theta -\left(1-\gamma_{1}\gamma_{2}\right)|{\left|{}_{r}D_{q}A\left(\theta c+\left(1-\theta \right)r\right)\right|}^{\mu }{}_{0}d_{q}\theta \\ & \leq \int_{0}^{1}\left|q\theta -\left(1-\gamma_{1}\gamma_{2}\right)\right|\left[h\left(\theta \right){\left|{}_{r}D_{q}A\left(c\right)\right|}^{\mu }+mh\left(1-\theta \right){\left|{}_{r}D_{q}A\left(\frac{r}{m}\right)\right|}^{\mu }\right]{}_{0}d_{q}t\\ & =\left(\int_{0}^{1}h\left(\theta \right)\left|q\theta -\left(1-\gamma_{1}\gamma_{2}\right)\right|{}_{0}d_{q}\theta \right){\left|{}_{r}D_{q}A\left(c\right)\right|}^{\mu }\\ & +m\left(\int_{0}^{1}h\left(1-\theta \right)\left|q\theta -\left(1-\gamma_{1}\gamma_{2}\right)\right|{}_{0}d_{q}\theta \right){\left|{}_{r}D_{q}A\left(\frac{r}{m}\right)\right|}^{\mu } \end{array}$$

and

(4) $$\begin{array}{cc} \int_{0}^{\gamma_{1}}| & {}_{r}D_{q}A\left(\theta c+\left(1-\theta \right)r\right){|}^{\mu }{}_{0}\;d_{q}t\\ & \leq \int_{0}^{\gamma_{2}}\left[h\left(\theta \right){\left|{}_{r}D_{q}A\left(c\right)\right|}^{\mu }+mh\left(1-\theta \right){\left|{}_{r}D_{q}A\left(\frac{r}{m}\right)\right|}^{\mu }\right]{}_{0}\;d_{q}\theta \\ & =\left(\int_{0}^{\gamma_{2}}h\left(\theta \right){}_{0}d_{q}\theta \right){\left|{}_{r}D_{q}A\left(c\right)\right|}^{\mu }+m\left(\int_{0}^{\gamma_{2}}h\left(1-\theta \right){}_{0}d_{q}\theta \right){\left|{}_{r}D_{q}A\left(\frac{r}{m}\right)\right|}^{\mu }. \end{array}$$

Applying power mean inequality, Lemma 1 and substituting inequalities (3) and (4) into the result, we have the following

$$\begin{array}{cc} |\gamma_{1} & \left[\gamma_{2}A\left(c\right)+\left(1-\gamma_{2}\right)A\left(r\right)\right]+\left(1-\gamma_{1}\right)A\left(\gamma_{2}c+\left(1-\gamma_{2}\right)r\right)-\frac{1}{c-r}\int_{r}^{c}A{\left(x\right)}_{r}\;d_{q}x|\\ & \leq \left(c-r\right)\left\{{\left(\int_{0}^{1}\left|q\theta -\left(1-\gamma_{1}\gamma_{2}\right)\right|{}_{0}\;d_{q}\theta \right)}^{1-\frac{1}{\mu }}\right.\\ & \times {\left({\left.{\left.\int_{0}^{1}\left|q\theta -\left(1-\gamma_{1}\gamma_{2}\right)\right|\right|}_{r}D_{q}A\left(\theta c+\left(1-\theta \right)r\right)\right|}_{0}^{r}\;d_{q}\theta \right)}^{\frac{1}{\mu }}\\ & \left.+\left(1-\gamma_{1}\right){\left(\int_{0}^{\gamma_{2}}1{}_{0}d_{q}\theta \right)}^{1-\frac{1}{\mu }}{\left(\int_{0}^{\gamma_{2}}{\left|{}_{r}D_{q}A\left(\theta c+\left(1-\theta \right)r\right)\right|}_{0}^{\mu }\;d_{q}\theta \right)}^{\frac{1}{\mu }}\right\}\\ \leq & \left(c-r\right)\left\{{\left[\int_{0}^{1}{\left|q\theta -\left(1-\gamma_{1}\gamma_{2}\right)\right|}_{0}\;d_{q}\theta \right]}^{1-\frac{1}{\theta }}\right.\\ & \times \left[\left(\int_{0}^{1}h\left(\theta \right)\left|q\theta -\left(1-\gamma_{1}\gamma_{2}\right)\right|{}_{0}d_{q}\theta \right){\left|rD_{q}A\left(c\right)\right|}^{\mu }\right.\\ & {\left.+m\left(\int_{0}^{1}h\left(1-\theta \right)\left|q\theta -\left(1-\gamma_{1}\gamma_{2}\right)\right|{}_{0}d_{q}\theta \right){\left|rD_{q}A\left(\frac{r}{m}\right)\right|}^{\mu }\right]}^{\frac{1}{\mu }}\\ & +\left(1-\gamma_{1}\right)\gamma_{2}^{1-\frac{1}{\mu }}\left[\left(\int_{0}^{\gamma_{2}}h\left(\theta \right){}_{0}d_{q}\theta \right){\left|{}_{r}D_{q}A\left(c\right)\right|}^{\mu }\right.\\ & \left.{\left.+{\left.\left.m\left(\int_{0}^{\gamma_{2}}h\left(1-\theta \right){}_{0}d_{q}\theta \right)\right|{}_{r}D_{q}A\left(\frac{r}{m}\right)\right|}^{\mu }\right]}^{\frac{1}{\mu }}\right\}. \end{array}$$

The proof is complete. □

When ${\left|{}_{r}D_{q}A\right|}^{\mu }$ with μ>1 is (h,m)-convex, then we obtain the following theorem.

**Theorem** **6.** *Assume that $A:\left[r,\frac{c}{m}\right]\to R$ is a continuous and q-differentiable function on $\left(r,\frac{c}{m}\right)$, such that 0≤r<c and m∈(0,1]. Assume that ${}_{r}D_{q}A$ is integrable on $\left[r,\frac{c}{m}\right]$, with 0<q<1, the inequality*

$$\begin{array}{c} \left|\gamma_{1}\left[\gamma_{2}A\left(c\right)+\left(1-\gamma_{2}\right)A\left(r\right)\right]+\left(1-\gamma_{1}\right)A\left(\gamma_{2}c+\left(1-\gamma_{2}\right)r\right)-\frac{1}{c-r}\int_{r}^{c}A{\left(x\right)}_{\mu }\;d_{q}x\right|\\ \leq \left(c-r\right)\left\{{\left(\int_{0}^{1}{\left|q\theta -\left(1-\gamma_{1}\gamma_{2}\right)\right|}_{0}^{\zeta }\;d_{q}t\right)}^{\frac{1}{\zeta }}\right.\\ \times {\left[\left(\int_{0}^{1}h\left(\theta \right)){}_{0}\;d_{q}\theta \right){\left|{}_{r}D_{q}A\left(c\right)\right|}^{\mu }+m\left(\int_{0}^{1}h\left(1-\theta \right){}_{0}\;d_{q}\theta \right){\left|{}_{r}D_{q}A\left(\frac{r}{m}\right)\right|}^{\mu }\right]}^{\frac{1}{\mu }}\\ +\left(1-\gamma_{1}\right)\mu^{\frac{1}{\zeta }}\left[\left(\int_{0}^{\mu }h\left(\theta \right){}_{0}\;d_{q}\theta \right){\left|{}_{r}D_{q}A\left(c\right)\right|}^{\mu }\right.\\ \left.{\left.+{\left.\left.m\left(\int_{0}^{\mu }h\left(1-\theta \right){}_{0}\;d_{q}\theta \right)\right|{}_{\mu }D_{q}A\left(\frac{r}{m}\right)\right|}^{\mu }\right]}^{\frac{1}{\mu }}\right\} \end{array}$$

*holds for all $\gamma_{1},\gamma_{2}\in \left[0,1\right]$ if ${|}_{r}D_{q}A{|}^{\mu }$ for μ>1, with $\mu^{-1}+\zeta^{-1}=1$, is (h,m)-convex on $\left[r,\frac{c}{m}\right]$.*

**Proof.**  Considering the Hölder inequality, the (h,m)-convexity for ${\left|{}_{r}D_{q}f\right|}^{r}$ and Lemma 1, we have

$$\begin{array}{c} \left|\gamma_{1}\left[\gamma_{2}A\left(c\right)+\left(1-\gamma_{2}\right)A\left(r\right)\right]+\left(1-\gamma_{1}\right)A\left(\gamma_{2}c+\left(1-\gamma_{2}\right)r\right)-\frac{1}{c-r}\int_{r}^{c}A{\left(x\right)}_{\mu }\;d_{q}x\right|\\ \leq \left(c-r\right)\left\{{\left(\int_{0}^{1}{\left|q\theta -\left(1-\gamma_{1}\gamma_{2}\right)\right|}_{0}^{\zeta }d_{q}\theta \right)}^{\frac{1}{\zeta }}\right.\\ \times {\left({\left.\left.\int_{0}^{1}\right|{}_{r}D_{q}A\left(\theta c+\left(1-\theta \right)r\right)\right|}^{\mu }{}_{0}d_{q}\theta \right)}^{\frac{1}{\mu }}\\ \left.+\left(1-\gamma_{1}\right){\left(\int_{0}^{\gamma_{2}}1_{0}^{\zeta }d_{q}\theta \right)}^{\frac{1}{\zeta }}{\left({\left.\int_{0}^{\gamma_{2}}\right|}_{r}D_{q}A\left(\theta c+\left(1-\theta \right)r\right)|{}_{0}\;d_{q}\theta \right)}^{\frac{1}{\mu }}\right\}\\ \leq \left(c-r\right)\left\{{\left(\int_{0}^{1}{\left|q\theta -\left(1-\gamma_{1}\gamma_{2}\right)\right|}_{0}^{\zeta }d_{q}\theta \right)}^{\frac{1}{\zeta }}\right.\\ \times \int_{0}^{1}\left[h\left(\theta \right){\left|{}_{r}D_{q}A\left(c\right)\right|}^{\mu }+mh\left(1-\theta \right){\left|{}_{r}D_{q}A\left(\frac{r}{m}\right)\right|}^{\mu }\right]{}_{0}d_{q}\theta \\ +\left(1-\gamma_{1}\right){\left(\int_{0}^{\gamma_{2}}1_{0}^{\zeta }d_{q}\theta \right)}^{\frac{1}{\zeta }}\int_{0}^{\gamma_{2}}\left[{\left.h\left(\theta \right)|{}_{r}D_{q}A\left(c\right)\right|}^{\mu }+{\left.\left.mh\left(1-\theta \right)\right|{}_{r}D_{q}A\left(\frac{r}{m}\right)\right|}^{\mu }\right]{}_{0}d_{q}\theta \\ \leq \left(c-r\right)\left\{{\left(\int_{0}^{1}{\left|q\theta -\left(1-\gamma_{1}\gamma_{2}\right)\right|}_{0}^{\zeta }d_{q}\theta \right)}^{\frac{1}{\zeta }}\right.\\ \times {\left[\left(\int_{0}^{1}h\left(\theta \right)){}_{0}d_{q}\theta \right){\left|{}_{r}D_{q}A\left(c\right)\right|}^{\mu }+m\left(\int_{0}^{1}h\left(1-\theta \right){}_{0}d_{q}\theta \right){\left|{}_{r}D_{q}A\left(\frac{r}{m}\right)\right|}^{\mu }\right]}^{\frac{1}{\mu }}\\ +\left(1-\gamma_{1}\right)\mu^{\frac{1}{\zeta }}\left[\left(\int_{0}^{\mu }h\left(\theta \right){}_{0}\;d_{q}\theta \right){\left|{}_{r}D_{q}A\left(c\right)\right|}^{\mu }\right.\\ \left.{\left.+{\left.\left.m\left(\int_{0}^{\mu }h\left(1-\theta \right){}_{0}d_{q}\theta \right)\right|{}_{\mu }D_{q}A\left(\frac{r}{m}\right)\right|}^{\mu }\right]}^{\frac{1}{\mu }}\right\}. \end{array}$$

□

## 4. Special Cases

This section discuses some special cases of Theorems 4–6 along with the relationship between the results reported therein.

**Corollary** **1.** *In Inequality (2) of Theorem 4,*

*i.*  *Choosing $h\left(\theta \right)=\theta^{\alpha }$ with α∈(0,1], we obtain Theorem 3.2 in [18].*
*ii.*  *Taking $\gamma_{1}=0$ and $\gamma_{2}=\frac{1}{1+q}$, we get the q-midpoint-like integral inequality for (h−m)-convexity*
(5) $$\begin{array}{c} \left|A\left(\frac{qr+c}{1+q}\right)-\frac{1}{c-r}\int_{r}^{c}A{\left(x\right)}_{r}d_{q}x\right|\leq M\left(\varsigma \right), \end{array}$$
  *where*
$$\begin{array}{c} M\left(\varsigma \right)=\left(c-r\right)\left\{\left[\int_{0}^{\frac{1}{1+q}}h\left(\theta \right)|q\theta |{}_{0}d_{q}\theta +\int_{0}^{1}h\left(\theta \right)\left|q\theta -1\right|{}_{0}d_{q}\theta \right.\right.\\ \left.-\int_{0}^{\frac{1}{1+q}}h\left(\theta \right)\left|q\theta -1\right|{}_{0}d_{q}\theta \right]\left|{}_{r}D_{q}A\left(c\right)\right|\\ +m\left[\int_{0}^{\frac{1}{1+q}}h\left(1-\theta \right)|q\theta |{}_{0}d_{q}\theta +\int_{0}^{1}h\left(1-\theta \right)\left|q\theta -1\right|{}_{0}d_{q}\theta \right.\\ -\int_{0}^{\frac{1}{1+q}}h\left(1-\theta \right)\left|q\theta -1\right|{}_{0}d_{q}\theta ]\left|{}_{r}D_{q}A\left(\frac{r}{m}\right)|\right\}. \end{array}$$
*iii.*  *Setting $\gamma_{1}=\frac{1}{3}$, $\gamma_{2}=\frac{1}{1+q}$ and m=1, we have q-Simpson-like integral inequality*
(6) $$\begin{array}{c} \left|\frac{1}{3}\left[\frac{qA(r)+A(c)}{1+q}+2A\left(\frac{qr+c}{1+q}\right)\right]-\frac{1}{c-r}\int_{r}^{c}A\left(x\right){}_{r}d_{q}x\right|\\ \leq \left(c-r\right)\left\{\left[\int_{0}^{\frac{1}{1+q}}h\left(\theta \right)|q\theta -\frac{q}{3(1+q)}|{}_{0}d_{q}\theta +\int_{0}^{1}h\left(\theta \right)\left|q\theta -\left(1-\frac{1}{3(1+q)}\right)\right|{}_{0}d_{q}\theta \right.\right.\\ \left.-\int_{0}^{\frac{1}{1+q}}h\left(\theta \right){\left|q\theta -\left(1-\frac{1}{3(1+q)}\right)\right|}_{0}d_{q}\theta \right]\left|{}_{r}D_{q}A\left(c\right)\right|\\ +m\left[\int_{0}^{\frac{1}{1+q}}h\left(1-\theta \right)|q\theta -\left(\frac{q}{3(1+q)}\right)|{}_{0}d_{q}\theta +\int_{0}^{1}h\left(1-\theta \right)\left|q\theta -\left(1-\frac{1}{3(1+q)}\right)\right|{}_{0}d_{q}\theta \right.\\ -\int_{0}^{\gamma_{2}}h\left(1-\theta \right)\left|q\theta -\left(1-\frac{1}{3(1+q)}\right)\right|{}_{0}d_{q}\theta ]\left|{}_{a}D_{q}A\left(\frac{r}{m}\right)|\right\}. \end{array}$$
*iv.*  *Choosing $\gamma_{1}=\frac{1}{2}$, m=1 and $\gamma_{2}=\frac{1}{1+q}$, we obtain the averaged midpoint-trapezoid-like integral inequality for (h−m)-convexity*
(7) $$\begin{array}{c} \left|\frac{1}{2}\left[\frac{qA(r)+A(c)}{1+q}+A\left(\frac{qr+c}{1+q}\right)\right]-\frac{1}{c-r}\int_{r}^{c}A\left(x\right){}_{r}d_{q}x\right|\\ \;\\ \leq \left(c-r\right)\left\{\left[\int_{0}^{\frac{1}{1+q}}h\left(\theta \right)|q\theta -\frac{q}{2(1+q)}|{}_{0}d_{q}t+\int_{0}^{1}h\left(\theta \right)\left|q\theta -\left(1-\frac{1}{2(1+q)}\right)\right|{}_{0}d_{q}\theta \right.\right.\\ \left.-\int_{0}^{\frac{1}{1+q}}h\left(\theta \right)\left|q\theta -\left(1-\frac{1}{2(1+q)}\right)\right|{}_{0}d_{q}\theta \right]\left|{}_{r}D_{q}A\left(c\right)\right|\\ +m\left[\int_{0}^{\frac{1}{1+q}}h\left(1-\theta \right)|q\theta -\left(\frac{q}{2(1+q)}\right)|{}_{0}d_{q}\theta +\int_{0}^{1}h\left(1-\theta \right)\left|q\theta -\left(1-\frac{1}{2(1+q)}\right)\right|{}_{0}d_{q}\theta \right.\\ -\int_{0}^{\mu }h\left(1-\theta \right)\left|q\theta -\left(1-\frac{1}{2(1+q)}\right)\right|{}_{0}d_{q}\theta ]\left|{}_{r}D_{q}A\left(\frac{r}{m}\right)|\right\}. \end{array}$$
*v.*  *Taking $\gamma_{1}=1$ and $\gamma_{2}=\frac{1}{1+q}$, we get the q-trapezoid-like integral inequality via (h−m)-convexity*
(8) $$\begin{array}{c} \left|\frac{qA(r)+A(c)}{1+q}-\frac{1}{c-r}\int_{r}^{c}A{\left(x\right)}_{r}d_{q}x\right|\\ \;\\ \leq \left(c-r\right)\left\{\left[\int_{0}^{\frac{1}{1+q}}h\left(\theta \right)|q\theta -\frac{q}{1+q}|{}_{0}d_{q}\theta +\int_{0}^{1}h\left(\theta \right)\left|q\theta -\frac{q}{1+q}\right|{}_{0}d_{q}\theta \right.\right.\\ \left.-\int_{0}^{\frac{1}{1+q}}h\left(\theta \right)\left|q\theta -\frac{q}{1+q}\right|{}_{0}d_{q}\theta \right]\left|{}_{r}D_{q}A\left(c\right)\right|\\ +m\left[\int_{0}^{\frac{1}{1+q}}h\left(1-\theta \right)|q\theta -\frac{q}{1+q}|{}_{0}d_{q}\theta +\int_{0}^{1}h\left(1-\theta \right)\left|q\theta -\frac{q}{1+q}\right|{}_{0}d_{q}\theta \right.\\ -\int_{0}^{\frac{1}{1+q}}h\left(1-\theta \right)\left|q\theta -\frac{q}{1+q}\right|{}_{0}d_{q}\theta ]\left|{}_{r}D_{q}A\left(\frac{r}{m}\right)|\right\}. \end{array}$$

**Remark** **1.** *As a especial case of Corollary 1, for h(θ)=θ, one can get some more results.*

*i.*  *In Inequality (5), choosing m=1, we get Theorem 3 which is established in [21].*
*ii.*  *In Inequality (6), if $q\to 1^{-}$, then we obtain Corollary 1 in [23].*
*iii.*  *In Inequality (7), if $q\to 1^{-}$, then we obtain Corollary 3.4 in [24].*
*iv.*  *In Inequality (8), taking m=1, we obtain Theorem 4.1 in [17].*

**Corollary** **2.** *In Theorems 5 and 6, if taking $\gamma_{2}=\frac{1}{1+q}$, we have the following particular cases*

*i.*  *Choosing $\gamma_{1}=0$, we obtain q-midpoint-like integral inequality for (h−m)-convexity.*
*ii.*  *Taking $\gamma_{1}=\frac{1}{3}$, we have q-Simpson-like integral inequality via (h−m)-convexity.*
*iii.*  *Choosing $\gamma_{1}=\frac{1}{2}$, we have the averaged midpoint-trapezoid-like integral inequality via (h−m)-convexity.*
*iv.*  *Taking $\gamma_{1}=1$, we get q-trapezoid-like integral inequality for (h−m)-convexity.*

**Remark** **2.** *If $\gamma_{2}=\frac{1}{1+q}$, when substituting $\gamma_{1}=\frac{1}{3},\gamma_{1}=0,\gamma_{1}=1$ and $\gamma_{1}=\frac{1}{2}$ in Theorems 5 and 6, we obtain the Simpson-like, the midpoint-like, the trapezoid-like and the averaged midpoint-trapezoid-like integral inequalities, respectively.*

## 5. Conclusions

In this study, variant types of *q*-integral inequalities including Simpson-like, midpoint-like, averaged midpoint-trapezoid-like types inequalities are generalized. It is important to note that the results obtained in this study in particular cases provide extensions to the results reported earlier in the literature. One advantage of our new inequalities is that they can be reproduced to several Riemann integral inequalities as well as classical integral inequalities for different classes of convexities, without proving each result independently. Since the study of Quantum calculus along with mathematical inequalities is still an active area of research, this work can be extended to coordinated convexity.

## Data Availability

Not applicable.
